# Simulation Methods and Validation Criteria for Modeling Cardiac Ventricular Electrophysiology

**DOI:** 10.1371/journal.pone.0114494

**Published:** 2014-12-10

**Authors:** Shankarjee Krishnamoorthi, Luigi E. Perotti, Nils P. Borgstrom, Olujimi A. Ajijola, Anna Frid, Aditya V. Ponnaluri, James N. Weiss, Zhilin Qu, William S. Klug, Daniel B. Ennis, Alan Garfinkel

**Affiliations:** 1 Department of Mechanical and Aerospace Engineering, University of California Los Angeles, Los Angeles, California, United States of America; 2 Department of Bioengineering, University of California Los Angeles, Los Angeles, California, United States of America; 3 Department of Medicine (Cardiology), University of California Los Angeles, Los Angeles, California, United States of America; 4 Department of Integrative Biology and Physiology, University of California Los Angeles, Los Angeles, California, United States of America; 5 Department of Radiological Sciences, University of California Los Angeles, Los Angeles, California, United States of America; Gent University, Belgium

## Abstract

We describe a sequence of methods to produce a partial differential equation model of the electrical activation of the ventricles. In our framework, we incorporate the anatomy and cardiac microstructure obtained from magnetic resonance imaging and diffusion tensor imaging of a New Zealand White rabbit, the Purkinje structure and the Purkinje-muscle junctions, and an electrophysiologically accurate model of the ventricular myocytes and tissue, which includes transmural and apex-to-base gradients of action potential characteristics. We solve the electrophysiology governing equations using the finite element method and compute both a 6-lead precordial electrocardiogram (ECG) and the activation wavefronts over time. We are particularly concerned with the validation of the various methods used in our model and, in this regard, propose a series of validation criteria that we consider essential. These include producing a physiologically accurate ECG, a correct ventricular activation sequence, and the inducibility of ventricular fibrillation. Among other components, we conclude that a Purkinje geometry with a high density of Purkinje muscle junctions covering the right and left ventricular endocardial surfaces as well as transmural and apex-to-base gradients in action potential characteristics are necessary to produce ECGs and time activation plots that agree with physiological observations.

## Introduction

The clinical management of cardiac arrhythmia is largely empirical due to our incomplete understanding of the underlying electrophysiology. Computational models of cardiac electrophysiology enable us to explore the arrhythmogenic impact of distinct causal factors, and to manipulate cardiac parameters that cannot be accessed experimentally. As described in [1], [2], cardiac electrophysiology can be modeled using a reaction-diffusion partial differential equation (PDE). The spatio-temporal variation in transmembrane potential results from two factors — cell-level ion channel-mediated ionic currents and current diffusion through extracellular gap junctions. Ionic currents at the myocardial cell level are described by non-linear ordinary differential equations (ODEs), which are then coupled via a diffusion PDE to describe the flow of current from cell to cell. The highly nonlinear ODEs, combined with the complex geometry and anisotropic conduction of the heart, make it impossible to solve the equations analytically, so numerical methods are required. The Finite Element Method (FEM) is widely used (e.g., [3]–[8]), primarily because it is the most flexible numerical technique for capturing the complex curved geometry of the heart. The use of FEM also allows to easily couple electrophysiology and mechanics simulations of the heart, since it is the method of choice for the mechanics problem. Furthermore, the numerical accuracy of FEM has been thoroughly verified through mathematical analysis and empirical benchmark testing on simple rectangular model geometries [9].

Recently, Pathmanathan and Gray [10] discussed the application of concepts of “Verification and Validation” to cardiac electrophysiology modeling. In many fields of science and engineering, there is a need to develop and codify “best practices for evaluating the reliability of computational models” [10], [11]. In fluid dynamics, solid mechanics, and other fields, rigorous standards have been developed to test models. This testing has two distinct dimensions: *verification* is concerned with showing that the model and its computational implementation have good convergence and error bounds in the calculation of “Quantities of Interest” [9], [10]; *validation*, on the other hand, is concerned with the relation of the model output to reality.

The criteria necessary for the verification of a model have been well accepted in the scientific community and several benchmark problems have been provided in the literature [9], [12] to assist with the model verification. The questions of validation are however more difficult to standardize. Great emphasis has been devoted to the validation of cell models but, until recently [13], less attention has been paid to the validation of whole heart multiscale models.

In the same spirit as Pathmanathan and Gray [10] and others in the cardiac modeling community [9], we have previously addressed the questions of verification of our computational framework in rectangular blocks [12]. Here, we aim to apply these verification criteria to the whole heart model and then focus on the question of model validation. Our goal is to investigate a series of objective criteria by which to evaluate whole heart models, specifically regarding the activation sequence, the generated ECG and the ability to model arrhythmia. Toward this purpose we define a unified 3D imaging model construction and an electrophysiology modeling framework for describing cardiac conduction. We first describe the extraction of geometry and microstructure from DT-MRI. Secondly, we provide a brief description of the tensor interpolation schemes used to integrate the experimental DT-MRI data into the numerical model. Further, we discuss the numerical solution of the monodomain equation for conduction and the inclusion of the Purkinje conduction system in our model. Finally, we describe the numerical computation of the ECG from the monodomain equation. We develop a series of validation criteria that we consider to be essential in the modeling of cardiac electrophysiology, including specific requirements on the ECG and time-activation plots.

## Materials and Methods

### Rabbit Biventricular Model Construction

DT-MRI provides detailed images of cardiac gross anatomy, and simultaneously provides quantitative microstructural (fiber orientation) information by estimating the local self-diffusion tensor (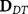) of water within each image voxel. DT-MRI was used to acquire anatomical and microstructural images from an *ex vivo*, healthy, female New Zealand White rabbit heart. Each subject was anesthetized with ketamine (10mg/kg i.v.) and xylazine (3mg/kg) and euthanized with an injection of B-euth (1mL/kg). The heart was excised, fixed in formalin, and imaged with a 7T Bruker Biospin MRI system using a 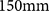 volume coil and a 3D RARE diffusion weighted pulse sequence (24 non-collinear diffusion gradient directions, 6 null directions, TR/TE  = 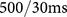, b-value  =  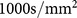, bandwidth  = 100 Hz per pixel, two-fold RARE acceleration, 0.5×0.5×0.75 mm resolution). Animal handling and care followed the recommendations of the Institutional Animal Care and Use Committee at the University of California, Los Angeles (UCLA) and the National Institutes of Health Guide for the Care and Use of Laboratory Animals. The animal protocol was approved by the UCLA Chancellors Animal Research Committee (Protocol #2008-161-12).

An in-house MATLAB (The Mathworks, Natick, MA) code was developed to process the diffusion weighted images and compute the diffusion tensors (this code is provided in S1 File). Corresponding to each imaging voxel, we computed both the eigensystem and the three invariants, i.e. trace, fractional anisotropy, and mode [14], of the diffusion tensor. The eigensystem decomposition of 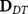 provides direct information about the local three-dimensional myofiber orientation (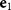, the primary eigenvector of 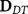) and the orientation of myolaminae (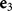, the tertiary eigenvector of 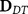) throughout the heart [15], [16].

Graph-based segmentation of DT-MRI using tensor invariant distances identified the myocardium [17]. A polyhedral mesh of the boundary surface was generated from the volume segmentation by the Marching Cubes algorithm using Paraview [18]. Surface mesh quality was improved using a windowed sinc filter [19], thereby reducing cell aspect ratios, smoothing sharp geometric features.

### Tensor Interpolation

In DT-MRI, data are acquired at lattice points within a 3D imaging volume, but numerical accuracy of FEM requires even finer mesh spacing, with nodes positioned between lattice points. Therefore, tensor field interpolation is a requirement. There are many methods to interpolate tensors including, but not limited to, nearest neighbor, Euclidean, log-Euclidean [20], geodesic-loxodrome [21], and linear-invariant tensor interpolation [22]. The advantage of geodesic-loxodrome and linear-invariant tensor interpolation is the monotonic and linear interpolation, respectively, of the tensor invariants (magnitude of isotropy, magnitude of anisotropy, and mode of anisotropy), which are intuitively related to salient microstructural features of the tissue [14]. The other tensor interpolation methods introduce microstructural bias, especially to the shape of the interpolated tensors [22].

**Figure 1:**
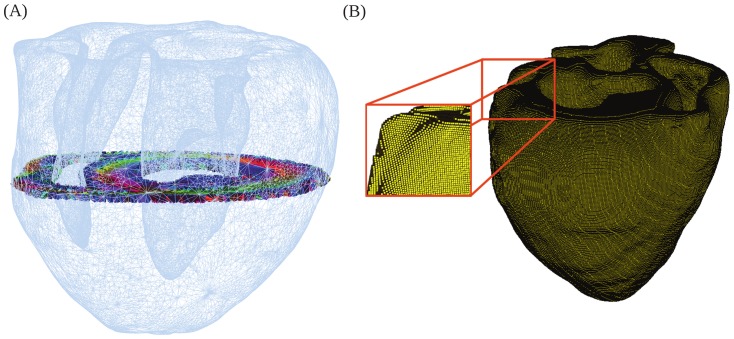
Tensor field and finite element mesh. (A) Short-axis slice of the linear invariant interpolated tensor field superposed on a coarsened surface mesh. (B) Hexahedral finite element mesh. The stair-stepped nature of the mesh is shown in the zoomed-in view of the model.

Only the orientation information, the eigenvectors of the interpolated diffusion tensors, was incorporated into the computational model, not the eigenvalues. This is because the directions of water diffusion correspond with the principal axes of the tissue microstructure, which in turn correspond to the directions of electrical propagation [23]. But there is no reason to expect that the magnitudes of electrical current diffusion are related to the magnitudes of water diffusion. For the electrical current diffusion magnitudes, we used eigenvalues in the ratio 4∶2∶1 [24], with the magnitudes scaled to reflect correct conduction velocities (see below). The interpolated tensor produced a value for the principal fiber direction at each integration point (Fig. 1A).

We compared different interpolation schemes (Geolox, log-Euclidean, Euclidean, and nearest neighbor) by considering their voltage predictions at each finite element node. We computed the Root Mean Squared Deviation (RMSD) between two interpolation schemes 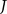 and 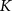 as 
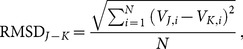
(1)where 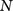 is the number of nodes 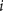 in the model, 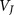 is the voltage computed with interpolation scheme 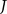 and 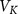 is the voltage computed with interpolation scheme 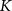.

### Finite Element Mesh Generation

Typical mesh generation software produces boundary conforming meshes, which have a distribution of element edge sizes. Numerical convergence studies [12] have concluded that elements must have edge lengths no larger than 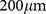 to guarantee adequate accuracy. Hence numerical efficiency is best achieved with meshes that have element sizes as close to 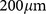 as possible. The straightforward way to do this would be a uniformly sized mesh, which will not be boundary conforming. The question we have here is one of validation: will such “stair-stepped” (Fig. 1B) non-boundary-conforming models give the right physiological results? We consider 2 different meshes, each constructed of hexahedral elements with trilinear interpolation (Table 1): (a) Stair stepped mesh with element edge size 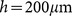, and (b) boundary conforming mesh with 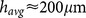 and 6% of the elements with edge size greater than 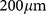. The stair-stepped (uniform) biventricular model is composed of 828,532 elements and 901,852 nodes (Fig. 1B) and the boundary conforming (non-uniform) mesh with 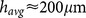 contains 991,705 elements and 1,055,649 nodes.

**Table 1:** Mesh statistics.

Mesh	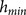	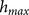	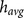
Stair Stepped 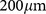	200	200	200
Boundary conforming with seed 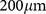	5	425	184

Edge length statistics for the non-boundary conforming stair stepped and boundary conforming meshes.

### Electrophysiology Modeling: Governing Equations

In the monodomain equation [1] the transmembrane voltage 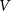 is governed by 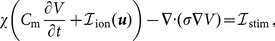
(2a)

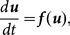
(2b)where 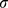 is the conductivity tensor, 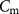 is the capacitance of a unit area of cell membrane, 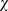 is the area of cell membrane per unit volume of tissue, and 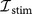 is the stimulus current. The conductivity tensor is related to the diffusion tensor as 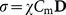, where 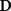 is the diffusion tensor. The set of internal cell state variables and voltage 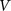 is represented by 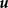, and its dynamic behavior is governed by the ordinary differential equations (ODEs) given by 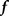. The ODEs couple back to the PDE through the ionic current 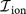. The single-cell ionic current is commonly modeled using a Hodgkin-Huxley framework [1], which describes the electrical activation potential of an excitable cell as the solution to a set of nonlinear ODEs. The identities of the ionic variables describing the gating of specific channels, as well as the choice of specific functional forms for 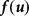, are determined according to experimental measurements of channel properties. We used the Mahajan *et al.* cell model [25] (see Cell Model Validation, below) with the parameters values defined in Table 2. We set 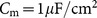 and the diagonal entries of 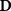 to 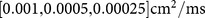 respectively along the fastest, medium, and slowest diffusion direction.

**Table 2:** Mahajan Cell Model Parameters.

Parameter	Description	Value
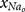	External Sodium Concentration	136.0 mM
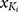	Internal Potassium Concentration	140.0 mM
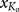	External Potassium Concentration	5.4 mM
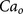	External Calcium Concetration	1.8 mM
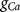	Strength of Ca Current Flux	182 mmol/(cm C)
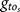	Peak 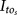 conductance	0.04 mS/µF
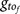	Peak 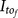 conductance	See Table 3
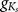	Peak 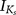 conductance	See Table 3
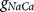	Strength of exchange current	0.84 µM/s
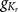	Peak 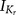 conductance	0.0125 mS/µF
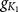	Peak 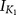 conductance	0.3 mS/µF
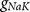	Peak 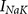 conductance	1.5 mS/µF
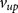	Strength of uptake	0.4 mM/ms
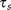	Submembrane-myoplasm diffusion time constant	4 ms
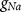	Peak 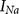 conductance	12.0 mS/µF
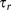	Spark lifetime	30.0 ms
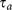	Non-junctional SR and dyadic junctional SR relaxation time	100.0 ms
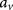	Release slope	11.3 ms 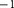
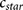	Threshold for steep release function	90 µM/1 cytosol
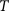	Temperature	308 K
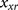	Universal Gas Constant	8.314 J mol 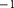 K 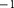
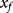	Faraday constant	96.485 C/mmol

Mahajan cell model parameters used in the electrophysiology simulations. The description of each parameter is taken from Mahajan *et al.* [25]

The monodomain equation was approximated using a finite element formulation [12], yielding the semidiscrete equations: 
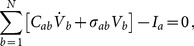
(3)where the capacitance matrix, conductivity matrix and ionic current vector 
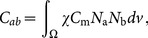
(4)

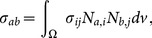
(5)

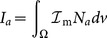
(6)are integrals computed over the region of space 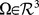 occupied by the biventricular model and 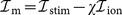. We employ ionic current interpolation and reaction-diffusion operator splitting, using row-sum lumped approximations of the capacitance matrices for both diffusive and ionic solution steps and a consistent mass matrix (the **C-LL** scheme described in Krishnamoorthi *et al.* [12]). The in-house C++ finite element code is provided at https://github.com/wsklug/UCLA_CMG.

### Purkinje Fiber and Purkinje Muscle Junction Modeling

Purkinje fibers physiologically form a specialized conduction system, which lies just beneath the endocardial surface. This special conduction network is isolated from the muscle except at its endpoints where it is connected to the ventricular endocardial surface at special sites called Purkinje-Muscle Junctions (PMJs). Modeling this network and its interaction with the muscle is crucial to build realistic ventricle models. The PMJ is bidirectional meaning that it transmits current from the conduction system to the myocardium and also from the myocardium retrogradely back to the conduction system.

Ten Tusscher and Panfilov [26] reviewed previous Purkinje models and highlight different approaches with respect to included anatomical details and strategies for exciting the myocardium. There is common consensus regarding the main anatomical features of the Purkinje network, i.e. a bundle of His that divides in to left and right bundle branches. To date, however, there remain challenges to incorporating accurate Purkinje networks into computational models, primarily because there are limited reports on imaging of the Purkinje network [27], [28].

The Purkinje network was manually incorporated into our computational model, as described in detail below, by assimilating the structure reported by Atkinson *et al.* [28]. The guidelines found in [27], [28] provide critical input about the placement of the Purkinje fibers. However, the precise segment lengths, distribution, and PMJ density play an important role in the activation of the model and hence the computed ECG. In general the conduction system has a single fiber emanating from the atrioventricular (AV) node, which then branches out into left and right bundles. The geometry of the conduction system beyond this is not well characterized and may vary significantly among individuals.

To model the Purkinje fibers we used 1D “bar” elements with Lagrangian isoparametric interpolation and linear shape functions 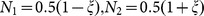 in terms of a parametric coordinate 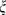. While computing the matrices in the finite element equations, we need the derivative of the shape function along global coordinate axis. Spatial (3D) gradients of the shape functions as needed for finite element matrices are computed using the chain rule 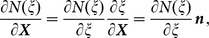


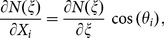
(7)where 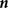 is the unit vector tangent to the fiber with the direction cosines as components. The scalar volume Jacobian is computed as the product of the length of the element with a specified cross sectional area 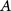 consistent with a circle of radius 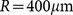.

To model a Purkinje muscle junction (PMJ) we begin connecting 1D elements to 3D myocardial nodes that lie within a search radius from a given terminal node in the Purkinje cable. Kirchhoff's law requires that the current out of the terminal Purkinje node match the total current into the PMJ, regardless of the number of branches from the terminal node. We choose to split this current evenly among the branches in the PMJ, attributing to the branches equal conductivities and equal fractions 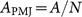 of the total cross-sectional area 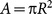. Conservation of current by Kirchhoff's law then gives
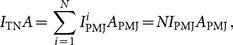
(8)where 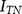 is the current density from the terminal node and 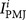 is the current density into the 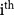 myocardium node. Shape function derivatives of the PMJ elements are computed using direction cosines as described above for the 1D Purkinje elements. To ensure simultaneous PMJ activation, PMJ branches are modeled with a fixed length of 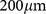. To eliminate the variation in the direction cosines of each 1D element in a PMJ, all the 1D elements are assigned the same direction cosine value. The value of direction cosines chosen is the value of the direction cosine of the 1D Purkinje element connected to the terminal node. Physiologically we would expect the PMJs to spread in the direction of the terminal node and hence this is an acceptable assumption.

The PMJ element thus takes on a standard isoparametric finite element formulation, albeit with non-standard shape functions constructed as shown. In this way the formulation retains complete mathematical consistency, without resorting to ad hoc “node-tying” constraint techniques. To attain proper source-sink matching at the 1D/3D interfaces, we selected PMJ myocardial nodes within a search radius equal to the Purkinje cross-sectional radius of 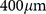. We verified that the formulation allows for successful bidirectional conduction (from Purkinje to myocardium, and in retrograde) across the 1D/3D interface. Retrograde activation of the Purkinje system is necessary to model Purkinje muscle reentry and the heart's response to Left or Right Bundle Branch Block (LBBB or RBBB) [29], although we do not carry those out here. If there is LBBB or RBBB, the QRS broadens, but not nearly as much as if there was only muscle-muscle conduction. Consequently, the activation of the heart in BBB must use retrograde activation of the Purkinje system.

**Figure 2:**
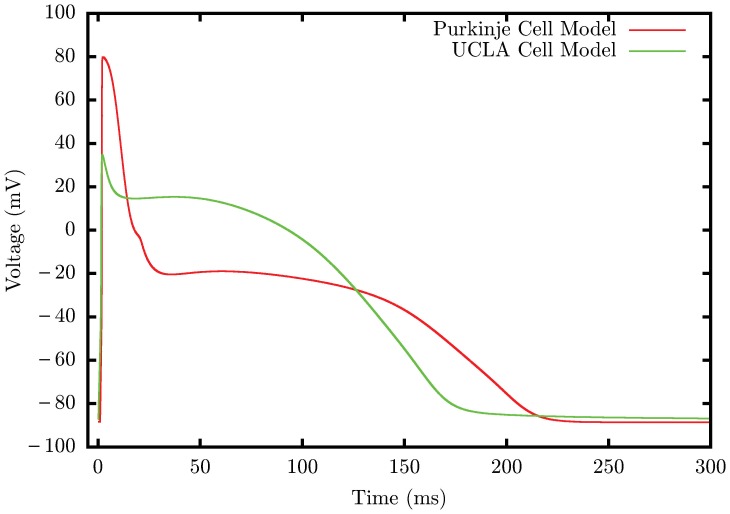
Action Potential (AP) plots of the Purkinje and normal UCLA cell model (Apex/Epi). The Purkinje AP shows a high upstroke velocity, a prominent early rapid repolarization, a negative plateau potential, an increased action potential duration, and spontaneous diastolic depolarization.

Activation of the Purkinje network was initiated with a stimulus of 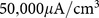 applied at the AV node for 5ms. For the Purkinje and PMJ elements we use the rabbit Purkinje cell model developed by Corrias *et al.* [30] (Fig. 2) and a diffusion 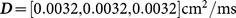. All simulations spanned two heartbeats, with a pacing interval of 400ms.

#### Purkinje Structure

In order to evaluate the importance of Purkinje geometry, we considered three different models.

**Figure 3:**
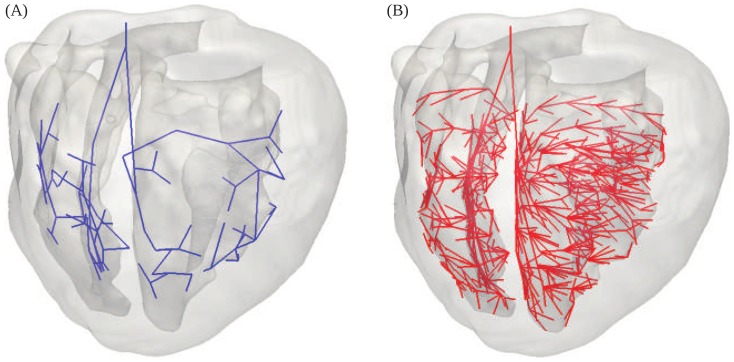
Purkinje models. (A) Low and (B) high PMJ densities.

- Low PMJ Model: This model has 54 PMJs. In both the LV and RV, a primary fascicle branches near the mid-septum, with the resulting branches traveling anteriorly and posteriorly. The posterior branch divides, continuing into the posterior base and the ventricular free wall, while the anterior branch terminates in the anterior base. There is a large region of the endocardial surface which is not connected to PMJs, and hence here cell-to-cell diffusion is the main mechanism of voltage propagation (Fig. 3A)
- High PMJ Model: This model has 514 PMJs and was generated by adding PMJs to the low PMJ model in order to reduce the dependence on cell-to-cell diffusion for voltage propagation. Here, the Purkinje network branches several times to thoroughly envelop the ventricles from the septum to the free wall in both the anterior and posterior directions.
- We also considered a model without a Purkinje structure, in which the heart was activated by simultaneous commanded activation of the endocardial surface.

### Cell Modeling and APD Gradients

The duration and morphology of the T wave in the ECG is determined by the sequence of repolarization in the heart. This sequence depends largely on the action potential duration (APD) gradients present in both the transmural and apex-to-base directions. These gradients arise from the heterogeneity of repolarizing currents within the heart, in particular the transient outward potassium current, 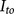, and the slow component of the delayed rectifier potassium current, 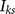.

**Figure 4:**
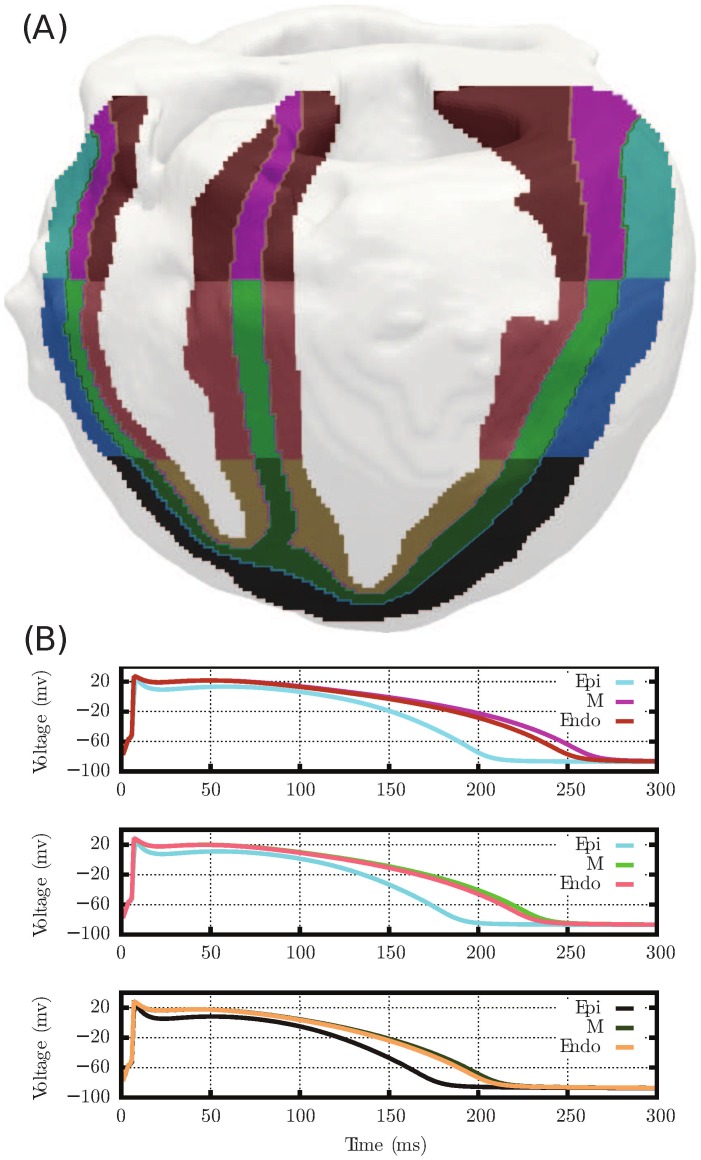
Apex-to-base and transmural APD gradients distribution. (A) Regional segmentation of the rabbit ventricular model and (B) corresponding action potentials. The colors of the heart segments match the color of the APD curves.

To incorporate these characteristics in our model, we divided the ventricle into transmural regions (endocardium, mid-myocardium or “M” cell, and epicardium), as well as apex-to-base regions (apex, mid, and base). This resulted in nine distinct regions. To each, we assigned a different variation of the Mahajan ventricular cell model [25] by altering the maximum conductance values for the 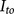 and 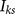 currents. These conductances (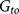 and 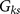 respectively) were defined in each region so as to produce the APD and current density gradients given in the literature (Table 3) (Fig. 4).

**Table 3:** Apex-to-base and transmural gradients.

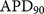 's(ms)	Apex	Center	Base
Epi	168	177	186
M	195	205	216
Endo	189	199	209

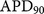
 and 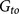, 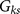 conductance values.

Transmurally, 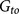 values were varied to match the data of Fedida *et al.* [31], who found the 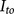 current density of endocardial cells to be 15% less than that of epicardial cells. 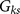 values were then adjusted to attain the APD gradient found by Idriss *et al.* [32], who reported the APD of endocardial cells and M cells to be 10% and 12% greater, respectively, than those of epicardial cells [32]. Mantravadi *et al.* [33] reported APD at the base to be 10% greater than at the apex. Because no data has shown that 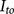 varies from apex to base, we only varied 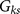 to achieve this (Table 3).

### Modeling of ECG

From a numerical model, the ECG output can be represented as [34]

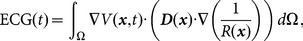
(9)where 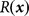 denotes the distance between any point 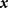 in the myocardial domain 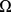 and the lead position, and 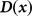 is the diffusion tensor. Only the elements defining the ventricles were included in the domain 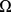 since the electrical mass of the Purkinje conduction system is negligible in comparison with the electrical mass of the ventricles.

#### ECG Lead Placement

**Figure 5:**
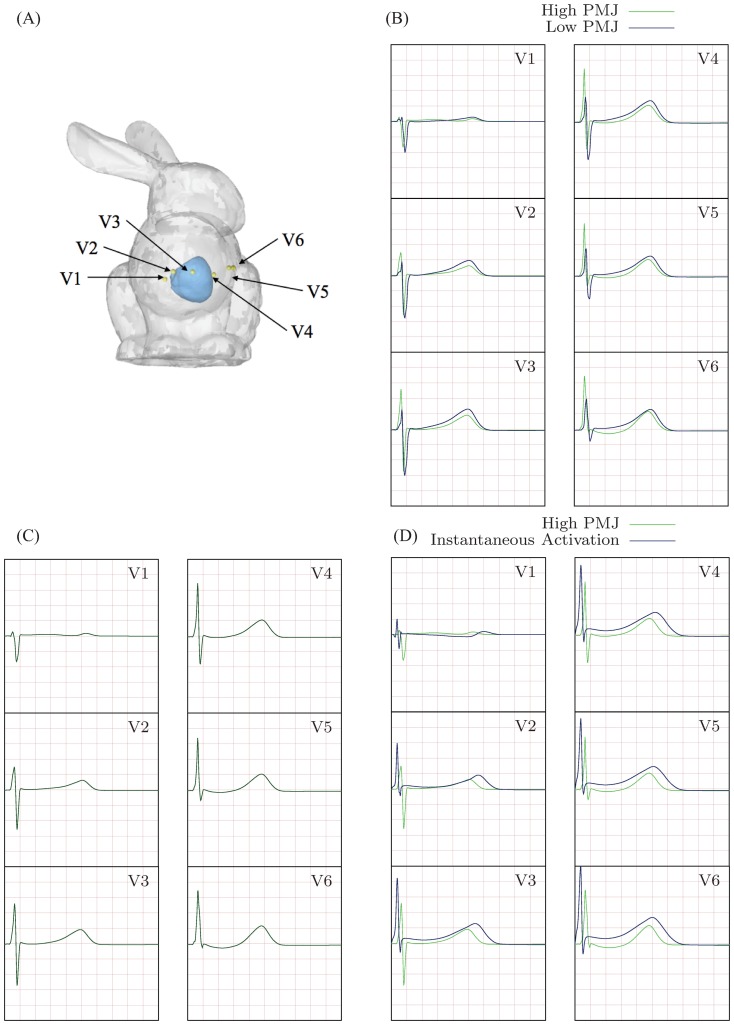
Six lead placement and corresponding ECGs computed using different activation models. (A) Six bipolar lead placement in the rabbit ventricular model. The model of the rabbit torso (Stanford Computer Graphics Laboratory) is shown to illustrate the lead positions but it is not part of the computational domain. (B) ECG obtained with the low PMJ density model shows slurring, fractionation and poor R-wave progression. (C) ECG obtained with the high PMJ density model shows the correct physiological features. (D) ECG obtained using instantaneous endocardial activation contains poor R-wave progression and slurring. Superimposed animated version of the ECG is provided as supplementary material (S1 Figure).

We calculated ECGs for the six precordial leads V1 to V6 positioned in specific positions on the chest wall. The six leads were placed according to the following guidelines: V1 - right sternal border; V2 - left sternal border; V3 - midway between V2 and V4; V4 - left midclavicular line; V5 - level with V4, left anterior axillary line; and V6 - level with V4, left mid axillary line. The placement of these leads is shown on a rabbit torso model (Fig. 5A); the torso itself was not part of the computational domain.

### Activation Maps

Following the pioneering work of Durrer [35], we constructed activation maps showing the temporal progress of the wavefront of activation. At each node in the domain 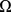, we linearly interpolated the voltage 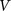 to calculate the time 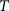 (from the activation of the AV node) at which a critical voltage 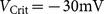 is reached:
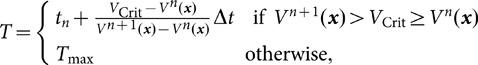
(10)where 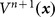 denotes the nodal voltage at time step 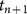, 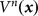 denotes the nodal voltage at time step 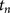, 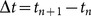.

### Validation Criteria

**Figure 6:**
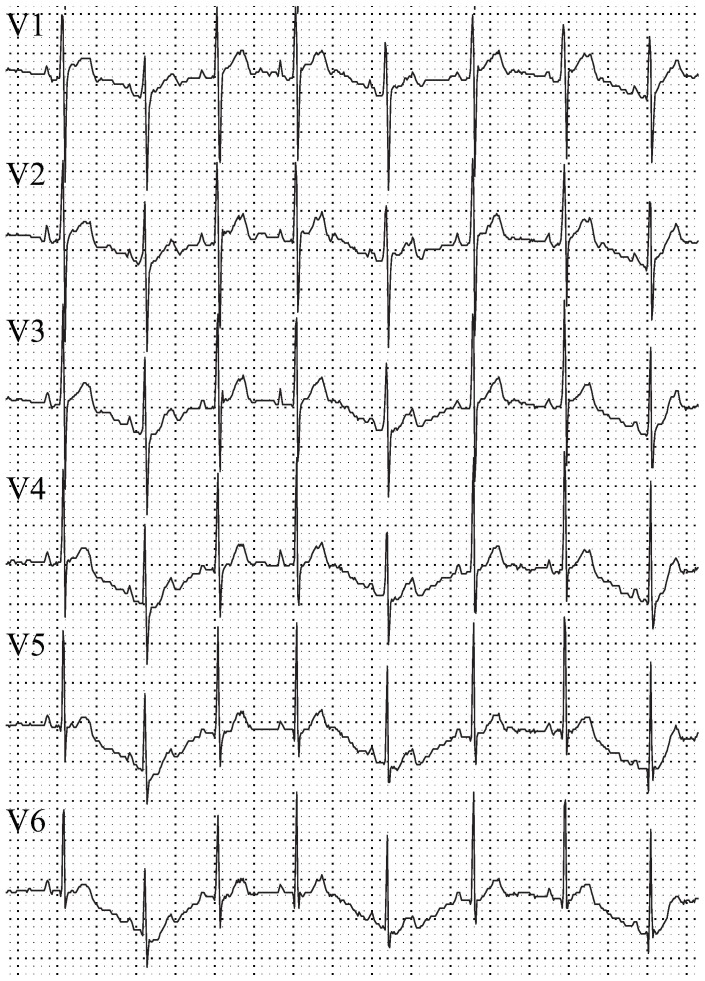
6-lead electrocardiogram of a normal adolescent White New Zealand male rabbit. The following defining aspects of the ECG are visible (5th validation criteria): fast QRS upstroke, no QRS fractionation, R-wave progressions from V1 to V6, positive T wave with longer upstroke than downstroke.

The challenges of model validation are not unique to the field of cardiac electrophysiology simulations [10], [11]. A limited number of measurable parameters, complicated geometries and the inability to validate all simulation results against experimental measurements make the task of validation even more cumbersome. In this context, we propose the following list of validation criteria regarding known features of cardiac electrophysiology that our model must capture correctly:

1. Cell Model. A ventricular cell model should reproduce correct Action Potential Durations (APDs) and Action Potential morphologies (rapid upstroke 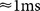, plateau phase, repolarization, epicardial “notch” where appropriate). The calcium transient should have the right waveform, latency, and duration. The cell model should also display marked APD shortening when paced at short intervals, the property called APD restitution. Capturing the correct action potential and calcium dynamics at rapid cardiac pacing is fundamental for modeling conditions such as tachycardia and fibrillation. We chose the Mahajan *et al.* cell model [25] because it meets these validation criteria.
2. Wavespeed: Conduction velocity should be 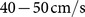. This wavespeed should not be sensitive to choices of numerical solution protocol, such as mesh density, numerical integration scheme, etc.
3. Electrical wavebreak: Excitation waves should only break up when encountering refractory tissue, not otherwise. Excitation waves must be free of artifactual wavebreak due to numerical methods.
4. Activation sequence: The septum should activate earliest, with earliest epicardial breakthrough in the RV, followed by the LV. There should be roughly simultaneous activation of the left and right ventricles [36].
5. Surface electrocardiograms: QRS duration in a rabbit should be less than 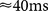 (Fig. 6) without fractionation or slurring (in humans, QRS duration is 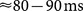). There should be R-wave progression, with R-waves becoming progressively more positive from V1 to V6 [37]–[39]. The T wave should be positive in all precordial leads and should have a longer rising than falling phase.
6. Response to stimuli: Multiple premature extrastimuli should initiate reentry. Reentry thus initiated should be unstable and degenerate into multiple repeated wavebreaks, simulating VF [40].

## Results

Unless otherwise stated, the primary model used to generate the following results includes the non-boundary conforming stair stepped mesh, the high density Purkinje model, the fiber orientations computed using the Geolox tensor interpolation, and the cell property gradients stated in Section “Cell Modeling and APD Gradients”.

Benchmark studies analyzing wave speed and wavebreak were previously described in [12] for rectangular geometries. We discuss these results in this section because of their importance in satisfying the validation criteria for full heart simulations.

### Wave Speed

The conduction speed of the wave of electrical depolarization in the rabbit heart is known from experimental observation to be 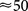
cm/s parallel to the fiber direction [41]. Previous modeling studies have shown that accurate recovery of the correct electrical wave speed in monodomain simulations is primarily an issue of convergence of the numerical solver [9], [12]. As noted above in Section “FE Mesh Generation”, we showed previously [12] in simple rectangular geometries that the numerical solution protocol used here yields wave speeds accurate to within 5% of the converged value so long as all elements in a mesh have edge lengths that are less than or equal to 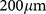. Based on these previous convergence studies [12] the error in conduction velocity will be 5% for the uniform mesh and as much as 20% for the boundary conforming mesh. It is especially important to note that meshes such as the boundary conforming mesh have a nonuniform distribution of element sizes, including some elements with edge lengths significantly greater than 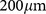, and so will yield artifactually nonuniform wave speeds, since larger elements conduct faster than smaller elements. As shown in Krishnamoorthi *et al.* [12] these errors can lead to artifactual curving or turning of electrical wavefronts, which will introduce artifacts into the activation sequence and ECG.

### Wavebreak

We have also shown previously [12] that the wave speed errors produced in unacceptably coarse (and especially nonuniform and coarse) meshes also can lead to artifacts in simulation of electrical wave break in heart tissue. Specifically in Krishnamoorthi *et al.* [12] we found that coarse meshes with elements of edge length greater than 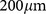 produced artifactual corners and jaggedness in spiral wavefronts, as well as spurious extinguishing of the activation.

### Activation Sequence

The sequence of electrical activation in the rabbit heart has been observed in electrical mapping experiments by Bordas *et al.* [36]. General features of the observed activation are

1. Early activation in the septum
2. Epicardial breakthrough in the RV first, followed by the LV
3. Roughly simultaneous activation of the left and right ventricles.

These features represent the criteria by which we evaluated the validity of our model. In particular we examined the impact on activation sequence of two model components: 1) tensor interpolation, and 2) Purkinje structure.

#### Tensor Interpolation

**Figure 7:**
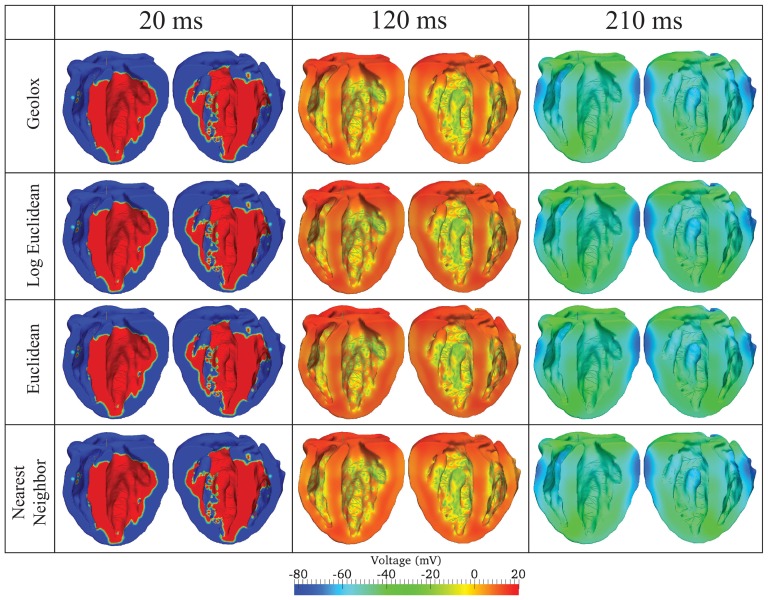
Voltage contour plots obtained with ventricular models using different tensor interpolation schemes. From top to bottom: Geolox, Log Euclidean, Euclidean, and Nearest Neighbor. Comparison of the voltage propagation is reported here at three time steps. From left to right: 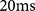, 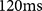, and 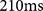.

Activation sequences for models constructed with Geolox, log-Euclidean, Euclidean, and Nearest Neighbor tensor interpolation schemes were computed over a single heartbeat. The voltage contours (Fig. 7) for all of the methods showed negligible differences throughout the beat cycle. RMS differences among the nodal voltages at every time step were at most 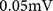 (data not shown). This maximum discrepancy was found between the models using Geolox and Nearest Neighbor interpolation. The maximum RMSD between Geolox and Euclidean interpolations was even smaller at 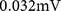. The maximum RMSD between Geolox and log-Euclidean interpolations was also 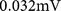. Among the three different Purkinje geometry models, the simulation with high PMJs shows minimal difference between different tensor interpolation schemes. The smallest differences in maximum RMSD were obtained when comparing Geolox, log-Euclidean, and Euclidean tensor interpolation methods, which show minimal differences in the primary eigenvector orientation [22]. However, although the Nearest Neighbor interpolation method leads to a relatively small RMSD due to the smoothing effects of diffusive coupling, it should be avoided due to large biases associated with interpolating the primary eigenvector [42]. If other tensor attributes (fractional anisotropy, etc.) were used in the computational model, then further evaluation would be needed.

#### Purkinje Structure

**Figure 8:**
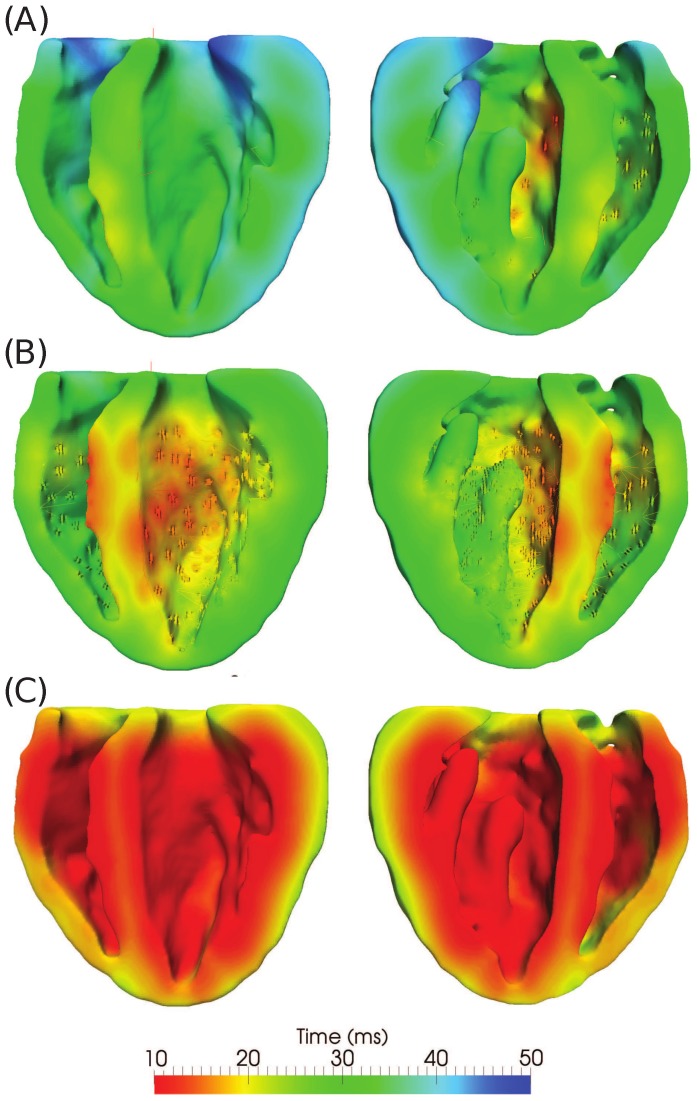
Comparison of activation contour plots obtained using different models of activation. (A) Low PMJ density model, (B) high PMJ density model, and (C) instantaneous activation of the LV and RV endocardial surfaces.

Voltage space-time histories were computed using each of the three activation models — (i) low PMJ, (ii) high PMJ, and (iii) instantaneous endocardial activation — to activate conduction in the uniform mesh with 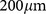 element edges, and processed to construct activation maps (Fig. 8 - raw data available as supplementary material in S1 Dataset) by eqn. 10. Geolox tensor interpolation was used in all simulations. The low PMJ model differed from the experimental observations, producing an overall delay in the initial septal activation (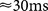). The complete activation of the myocardium was slower, with the basal region activated more than 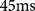 after stimulus of the AV node. The high PMJ model showed better agreement with experimental mapping results, showing synchronous activation of the LV and RV endocardium, and earlier septal activation (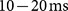). The high PMJ model also yielded complete depolarization of the myocardium by about 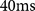, consistent with experiments [36]. The activation movie corresponding to the high PMJ model is provided as supplementary material (S1 Movie).

The model with instantaneous activation of the RV and LV endocardium shows activation comparable to the high PMJ model: it produces synchronous activation of the LV and RV and a rather rapid depolarization of the entire myocardium (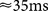). Overall, the activation patterns from the high PMJ and the instantaneous activation models match best with experimental data. However, the ECGs do not agree (see below and Fig. 5).

### Electrocardiograms

Based on the activation sequence results, we set aside the effect of tensor interpolation and focused instead on the effects of the Purkinje structure on the ECG. We calculated the ECG (Fig. 5 - raw data and animated files provided as supplementary material in S2 Dataset and S1 Figure) using eqn. 9.

#### QRS Duration

Since the QRS duration recorded at each individual ECG lead is slightly different, we report an average QRS width and its standard deviation for each ECG obtained with different activation models. The average width of the QRS over the 6 leads was 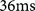 (standard deviation  =  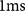) for the low PMJ model, 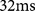 (standard deviation  =  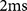) for the high PMJ model, and 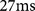 (standard deviation  =  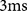) for the instantaneous activation model. The high PMJ and instantaneous models both produce a narrower QRS duration than the low PMJ model and this is consistent with the rapid depolarization observed in the activation maps. Conversely the wider QRS indicates that the low PMJ model is slower to depolarize.

#### QRS Morphology

The low PMJ model shows a slow stunted rise (referred to as slurring) in the R-wave in all leads, especially V1 through V3, and in the beginning of the ST segment for leads V1 through V5 (Fig. 5B). The high PMJ model does not show slurring in the R-wave, and we do not observe any fractionation in the ECG (Fig. 5C). Fractionation and slurring are not present in a healthy rabbit ECG (Fig. 6). The instantaneous activation model shows slurring, particularly in leads V2 and V3 (Fig. 5D).

#### R-Wave Progression

The low PMJ and instantaneous activation models lead to poor R-wave progression since the ratio between the amplitudes of the R and S waves is not correct. For example, in lead 5 of the low PMJ model computed ECG, this ratio is approximately one-to-one while in the experimental rabbit ECG, we notice that the R-wave amplitude is much larger than that of the S-wave (Fig. 5B). In the instantaneous activation model, the amplitude of the S-wave is much smaller than that of the R-wave in leads V2 through V6 (Fig. 5D). The high PMJ model produces a physiologically correct R-wave progression and has the correct ratio of amplitudes (when compared to the experimental rabbit ECG Fig. 6) through the six precordial leads (Fig. 5C).

#### T-Wave Morphology

The inclusion of the APD gradients (longer APDs in the endocardium and base than in the epicardium and apex, respectively) in all three models creates a repolarization wave traveling from the apical epicardium to the basal endocardial surface. Because this is a repolarization vector moving away from the leads, it generates an upright T-wave in all leads. Both transmural and apex-to-base gradients are required to reproduce the T-wave morphology characteristic of the ECG of a healthy heart. The presence of only apex-to-base or transmural gradients produces a low amplitude T-wave and the presence of apex-to-base gradients alone leads to symmetric T-waves (see supplementary material S1 Figure); neither are observed in the healthy heart ECG.

#### Sensitivity of the Electrocardiogram to Boundary Surface Definition

**Figure 9:**
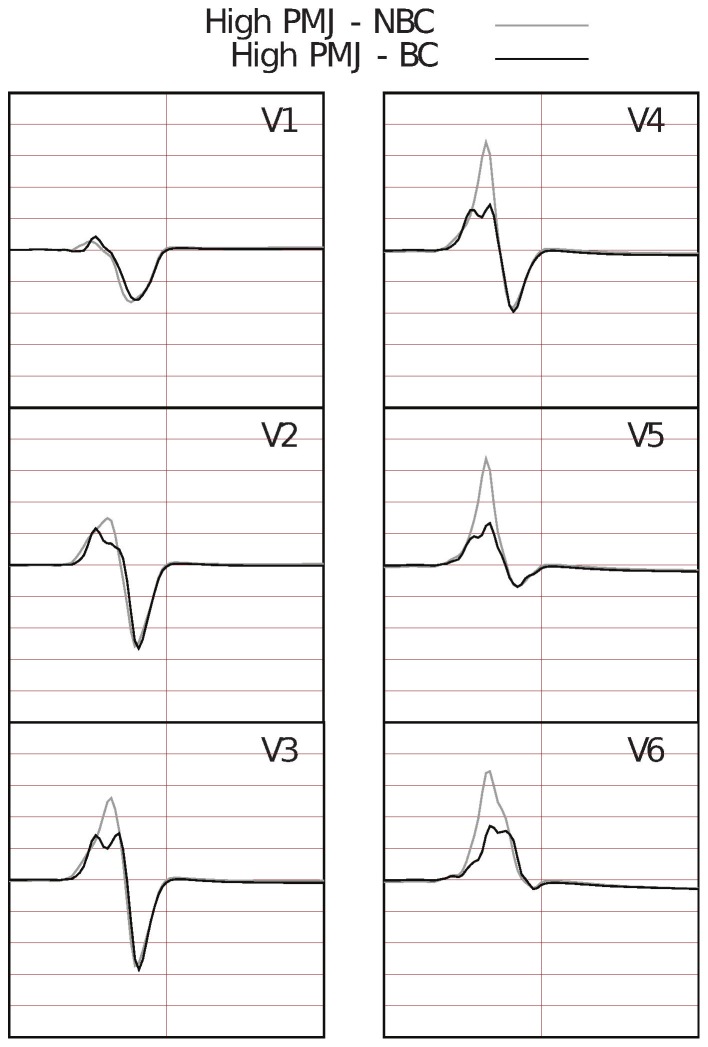
Six Lead ECG focusing on the QRS wave for non-boundary conforming (NBC) and boundary conforming (BC) models with mesh size 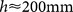. ECG computed using the boundary conforming model shows severe fractionation and incorrect R-wave progression.

As discussed above in Sections “FE Mesh Generation” and “Wave speed”, the mandates for computational efficiency and wave speed accuracy together suggest the use of finite element meshes that have distributions of element sizes as close to uniform as possible, with no element exceeding 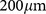 in edge length. However, perfectly uniform hexahedral meshes (all elements are identical cubes) will necessarily have non-smooth stair-stepped boundaries that do not conform to the smooth epi- and endocardial surfaces of the heart. To quantify the effect of this non-conforming boundary approximation, we evaluate the time activation plots and the ECGs obtained using the boundary conforming (nonuniform) and stair-stepped (uniform) meshes described in Table 1. In these analyses we use the high PMJ model. While the time activation plots (not shown here) are very similar, the QRS waves in the ECG (Fig. 9) show significant differences. The uniform (stair-stepped) mesh produces physiologically correct results, without slurring and fractionation and with a correct R-wave progression. On the other hand, the boundary conforming mesh (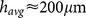 and 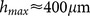) leads to severe fractionation and poor R-wave progression in the ECG.

### Induction of Reentry

**Figure 10:**
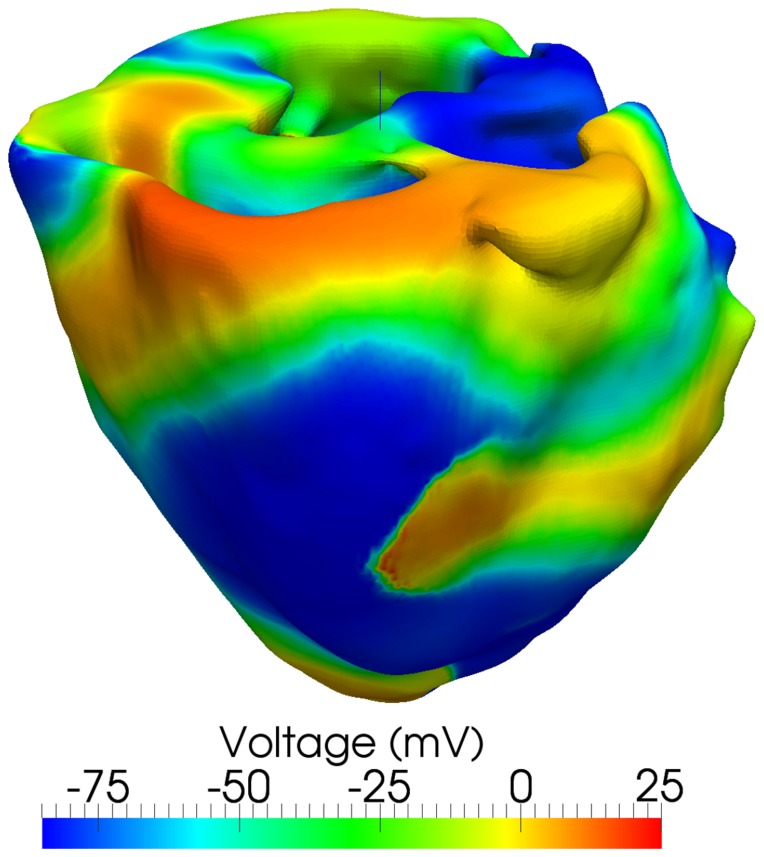
Sustained wave breakup and chaotic meandering during simulated Ventricular Fibrillation (VF). VF was induced using an S1-S2 protocol with the second stimulus applied between 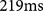 and 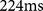. The voltage contour plot is shown at 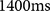.

In a normal heart, one strong second stimulus (S2) delivered in the refractory tail of the previous wave (stimulated by a normal S1) can initiate reentry [40], [43]–[45]. Under pro-arrhythmic conditions, once initiated, reentry is unstable, and, within seconds, breaks up into a multi-wave chaotic state in which wavefronts are being continually generated and extinguished [40], [43], [46]. In small electrical substrates such as a rabbit heart, sustained chaotic wave break-up can be induced by decreasing conduction velocity [43]. We observed sustained fibrillation in our primary model (described in the beginning of the “Results" section) with 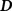 reduced by 25%. Application of a 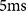 stimulus of 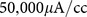 in a 60-degree wedge-shaped region of the LV freewall covering half the height of the heart (S2) at 219ms following normal Purkinje activation (S1) initiated a reentrant wave. This wave broke up into a sustained multi-wave state representing ventricular fibrillation (Fig. 10 and voltage propagation video provided as supplementary material as S2 Movie and S3 Movie).

Previously, Berenfeld and Jalife [47] have shown that retrograde activation of the Purkinje system in a ventricular model is necessary in initiating and stabilizing polymorphic tachycardia. In our study, we show that during ventricular fibrillation, the Purkinje network is retrogradely activated starting in the right ventricle at time 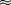 1s (see supplementary material S2 Movie and S3 Movie). From a physiological standpoint, this model prediction suggests that the Purkinje network may play a role in the propagation of sustained VF after its onset. Moreover, from a modeling point of view, this result indicates how a correct model of the Purkinje system allowing for retrograde activation is important even in absence of bundle branch block

## Discussion

The use of models to investigate the electrophysiology of the heart must be preceeded by verification and validation. Verification is concerned with answering the question: are we solving the equations correctly? Validation asks: are we solving the correct equations?

While we emphasize the necessity of verification and validation, we acknowledge that the experimental parameters governing electrophysiology vary among different subjects and different sets of experiments. This experimental uncertainty may have on the results an effect greater than the accuracy sought through verification and validation. Nevertheless, an inherently inaccurate model impairs our capability to scientifically investigate any phenomena, since the conclusions drawn from such models with incorrectly solved equations cannot be trusted. On the other hand, only an accurate model enables us to investigate the effect of parameter variability. This crucial analysis falls under a third category alongside verification and validation: model sensitivity and uncertainty quantification. However, in this work, we primarily focus on proposing a series of important verification and validation criteria and in applying those to our model.

In the evaluation of our model, the first question focuses on verification. Only, a correct solution to the equations leads to an accurate wavespeed and no artificial wavebreak. The choice of finite element parameters have a significant impact on satisfying these verification criteria. Specifically, the *maximum* element edge length *- not the average -* is a critical indicator of whether to expect mesh-related artifacts in the physiological results. Even a few large elements will produce artifactually high local wave speeds, leading to distortion of wavefronts, as shown in [12], [48].

The second criterion in evaluating our model concerns validation. A correct physical model will produce physiologically accurate activation sequences. Both a high density Purkinje network and a simultaneous activation of the endocardium satisfy this criterion. In contrast, a low density Purkinje network is inadequate in activating the heart correctly. In evaluating the validity of a high density Purkinje network and the simulaneous endocardial activation strategies, the ECG is a more sensitive criterion. The ECG reveals that instantaneous endocardial activation produces slurring and poor R-wave progression whereas the high density Purkinje system leads to the correct QRS morphology. Furthermore, the inclusion of a Purkinje network is more physiologically realistic and enables, for example, the study of retrograde activation as occurs in bundle branch block conditions.

Another validation criterion contained within the ECG concerns the T-wave morphology. The cause of the T-wave is repolarization dispersion [49]. Although there is no consensus over the relative contributions of apex-to-base versus transmural cell properties gradients [50], we use both in our model and recover a physiologically correct T-wave morphology. Our findings that transmural gradients are required to produce an asymmetric T-wave agree with the discussion and ECGs shown in figure four of [49]. Further, we found that both apex-to-base and transmural gradients are necessary for producing the correct height of the T-wave. In this regard, Keller *et al.* observed that the presence of both transmural and apex-to-base cell properties gradients lead to the best agreement between computed and measured ECG. Although the simulation setup in our study and the research of Keller *et al.* was different (e.g., 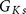 gradients and cell model are different) both studies suggest the necessity of combining transmural and apex-to-base gradients to achieve physiologically accurate ECGs.

The analysis of the ECG also allows us to compare stair-stepped and boundary conforming meshes. We carry out this comparison using meshes with equal average mesh size of 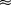 200 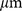, resulting in approximately the same number of elements and nodes, and therefore roughly equal computational cost. Both boundary conforming and non-boundary conforming models produce similar activation plots but only the stair stepped mesh produces a physiologically correct ECG. We believe that the physiologically incorrect ECG obtained using the boundary conforming mesh is due to the presence of large elements (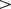 200 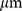) causing artifactual wave speed. Therefore, although we intended it for validation, the ECG also provides an additional tool for verification, showing the effects of large numerical errors. Based on the analyses and comparisons presented herein, it appears that stair stepped models satisfy both verification and validation criteria. However, a careful study of the effect of boundary conformity on the accuracy of the results is warranted, including a comparison between boundary and non-boundary conforming models with equal maximum element size (boundary conforming meshes with element size 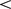 200 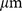 lead to a significant increase in computational cost). Although this is an important subject of future research, it falls outside the scope of the study presented here.

A last validation criterion that we consider is the ability to induce wave reentry and ventricular fibrillation. Our model, based on physiologically accurate cell models, microstructure, and activation mechanisms, is capable of capturing this phenomenon. This result suggests that our model is suitable for analyzing normal as well as abnormal conditions and may be applied not only to reproduce known phenomena but also for predictive studies.

Naturally the list of validation criteria is not static but broadens with new experimental findings and enhanced numerical capabilities. Herein, we have presented a select series that we consider to be important.

A verified and validated model is an essential starting point if we want to quantify the uncertainty related to parameter variability. Although a thorough sensitivity analysis of our framework is outside the scope of this paper, the construction of our model required the evaluation of different tensor interpolation schemes. When quantifying the effect of these schemes on the activation sequence of the full heart, we found that the various tensor interpolation methods were not distinctly different. This is likely because the Geodesic-loxodrome, Euclidean, and log-Euclidean interpolation methods have a similar accuracy in recapitulating the myofiber orientation (e.g. fiber angle) [51]. Although the activation sequence looks comparable, it should be noted that Nearest Neighbor interpolation has an increased bias in approximating the primary eigenvector. Furthermore, Geodesic-loxodrome and linear invariant schemes are more accurate in interpolating tensor shape attributes (fractional anisotropy, etc.) than Euclidean and log-Euclidean methods [22]. For example, if the electrical conductivities of the tissue were to be scaled by the eigenvalues of the water diffusion tensor obtained from DT-MRI, the use of Geodesic-Loxodrome or linear invariant tensor interpolation methods is recommended due to the better recovery of tensor shape. Whether a relationship exists between the magnitude of the electrical conductivity and the diffusion tensor shape in cardiac tissue is currently unreported, but has been explored in brain tissue [52].

Our focus here has been on a rabbit heart anatomical model, with models of the electrophysiology of rabbit myocytes and Purkinje cells. We have validated this model against rabbit ECGs and other physiological properties of the rabbit. However, obviously, the long-term interest of any such modeling has to be on the human heart and its properties. It is important to note that all of the validation criteria we are using here would also pertain to a human heart, and therefore our criteria are valid beyond rabbit-specific modeling. The analogy of rabbit to human, and the use of the rabbit as a model for human ventricular physiology, is also in keeping with the dynamical similarity of rabbit cardiac electrophysiology to human. Indeed, using scaling arguments, Panfilov [53] has argued that the “effective size” of the human heart, scaled by electrophysiology properties, is closer to the rabbit than to either pig or dog.

We conclude by pointing out some limitations in our model that we aim to address in the future development of our framework. Mainly, we are concerned with improving the calculation of the ECG, which has proven to be the most sensitive tool in our model validation. In this regard, accounting for a torso model and a bidomain formulation will allow us to compute a more physiologically accurate ECG, which will include the Einthoven leads. Furthermore, we recognize that we did not consider the electro-mechanical coupling in our cell model, e.g., stretch-activated channels. Another improvement of our model consists in varying continuously the cell properties transmurally and from apex to base instead of having nine distinct cell regions. While nine discrete regions are sufficient to obtain correct APD gradients and T-wave morphology, the cell properties in the myocardium are likely to vary continuously and the presence of discrete boundaries (discontinuities) may, in principle, trigger artifactual wave breaks. Although no spurious wave break was observed in any of the simulations conducted in this study, this potential source of artifacts needs to be considered and, if possible, eliminated using continuously varying cell properties.

Although numerous improvements in the calculation of the ECG are possible, these do not diminish its importance. In this work, we have highlighted the necessity and sensitivity of the ECG in the validation of an electrophysiology model.

## Supporting Information

S1 Figure **ECG Comparison.** Differences in the computed ECG using the three activation models and the different gradients.
(ZIP)

S1 Dataset **Activation Paraview visualization (.vtu format) files for high-, low-PMJ model, and instantaneous activation model.**
(ZIP)

S2 Dataset **ECG raw data for the six lead ECG.** Data was obtained at every millisecond.
(ZIP)

S1 File **Code for computing tensor interplation data.**
(ZIP)

S1 Movie
**Normal activation.** Time 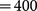
ms.(MP4)

S2 Movie
**Four-view ventricular fibrillation.** Time 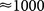
ms.(MP4)

S3 Movie
**Open-view ventricular fibrillation.** Time 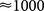
ms.(MP4)
