# Supplementary figures and images for: Simulation Methods and Validation Criteria for Modeling Cardiac Ventricular Electrophysiology
Source: PLoS One. 2014 Dec 10;9(12):e114494. doi: 10.1371/journal.pone.0114494 (PMC4262432; doi:10.1371/journal.pone.0114494)

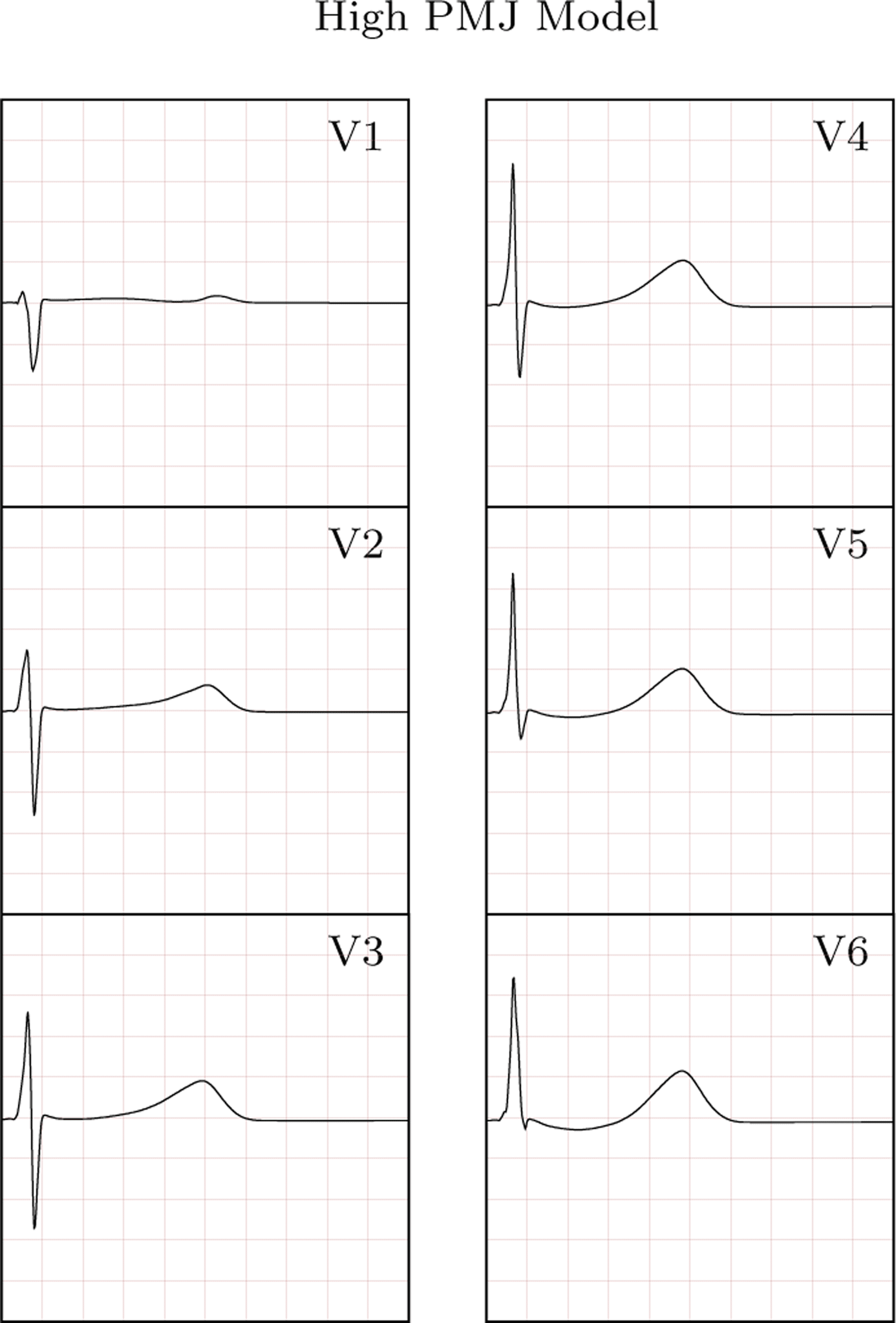

Supplement: S1 Figure — ECG Comparison. Differences in the computed ECG using the three activation models and the different gradients. (ZIP) [file pone.0114494.s001.zip › Figure S1/ECG_ActivationModel_comparison.gif]

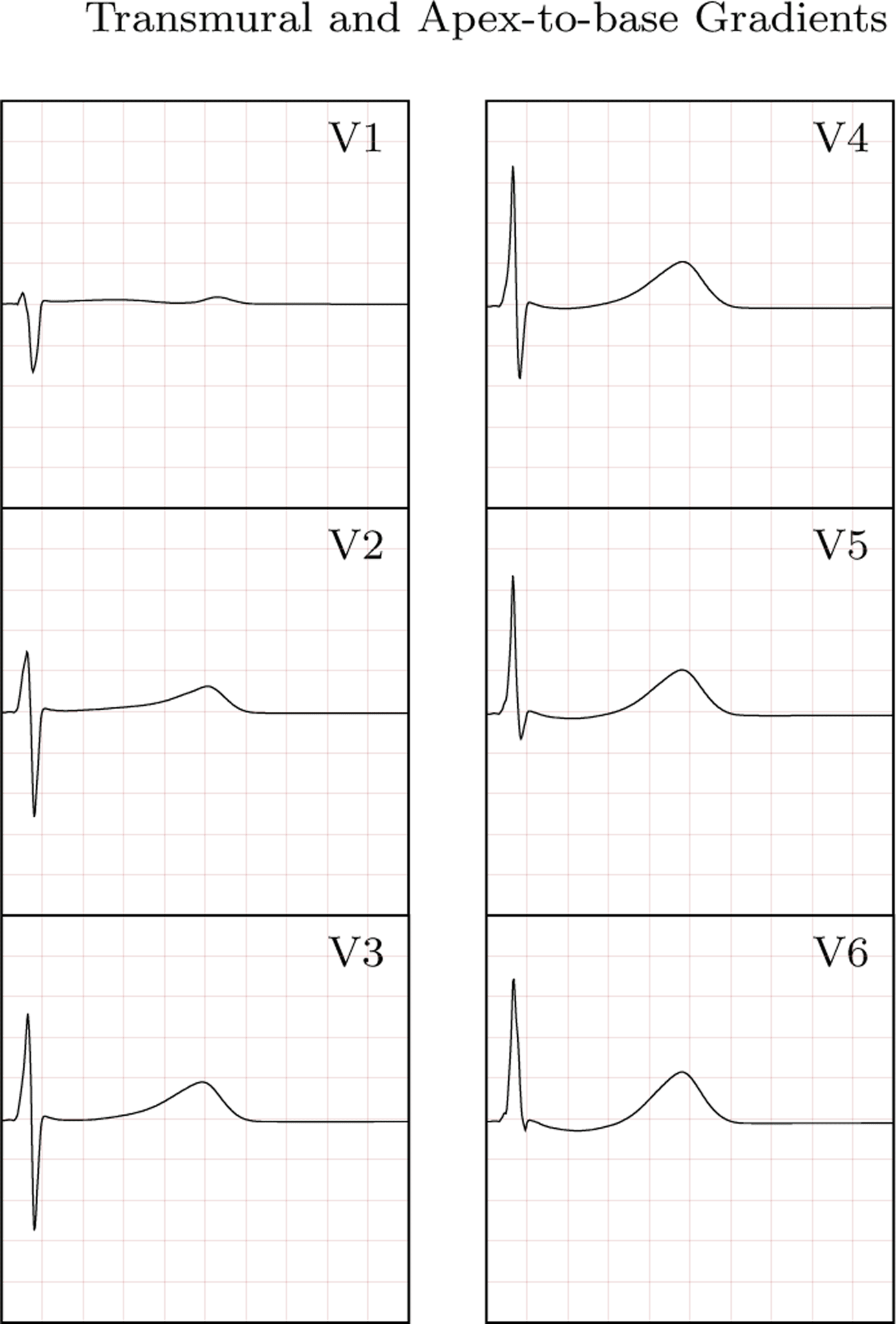

Supplement: S1 Figure — ECG Comparison. Differences in the computed ECG using the three activation models and the different gradients. (ZIP) [file pone.0114494.s001.zip › Figure S1/ECG_gradients_comparison.gif]
